# Development of a Nucleic Acid Extraction Procedure for Simultaneous Recovery of DNA and RNA from Diverse Microbes in Water

**DOI:** 10.3390/pathogens4020335

**Published:** 2015-05-26

**Authors:** Vincent R. Hill, Jothikumar Narayanan, Rachel R. Gallen, Karen L. Ferdinand, Theresa Cromeans, Jan Vinjé

**Affiliations:** 1 Centers for Disease Control and Prevention, National Center for Emerging and Zoonotic Infectious Diseases, Division of Foodborne, Waterborne, and Environmental Diseases, 1600 Clifton Road NE, Mailstop D-66, Atlanta, GA 30329, USA; E-Mails: jin2@cdc.gov (J.N.); rrgallen@gmail.com (R.R.G.); 2 Centers for Disease Control and Prevention, National Center for Immunization and Respiratory Diseases, Division of Viral Diseases, Atlanta, GA 30329, USA; E-Mails: karen.ferdinand@abbott.com (K.L.F.); trc1@cdc.gov (T.C.); ahx8@cdc.gov (J.V.)

**Keywords:** drinking water, water testing, nucleic acid, DNA, RNA, extraction, purification, PCR facilitators

## Abstract

Drinking and environmental water samples contain a diverse array of constituents that can interfere with molecular testing techniques, especially when large volumes of water are concentrated to the small volumes needed for effective molecular analysis. In this study, a suite of enteric viruses, bacteria, and protozoan parasites were seeded into concentrated source water and finished drinking water samples, in order to investigate the relative performance of nucleic acid extraction techniques for molecular testing. Real-time PCR and reverse transcription-PCR crossing threshold (CT) values were used as the metrics for evaluating relative performance. Experimental results were used to develop a guanidinium isothiocyanate-based lysis buffer (UNEX buffer) that enabled effective simultaneous extraction and recovery of DNA and RNA from the suite of study microbes. Procedures for bead beating, nucleic acid purification, and PCR facilitation were also developed and integrated in the protocol. The final lysis buffer and sample preparation procedure was found to be effective for a panel of drinking water and source water concentrates when compared to commercial nucleic acid extraction kits. The UNEX buffer-based extraction protocol enabled PCR detection of six study microbes, in 100 L finished water samples from four drinking water treatment facilities, within three CT values (*i.e.*, within 90% difference) of the reagent-grade water control. The results from this study indicate that this newly formulated lysis buffer and sample preparation procedure can be useful for standardized molecular testing of drinking and environmental waters.

## 1. Introduction

Molecular analytical techniques are valuable tools for evaluating the microbial quality of water. For drinking water systems, molecular testing can provide rapid, sensitive, and specific data for evaluating source water quality, water treatment performance, and distribution system integrity. The most common approach for molecular detection of waterborne microbes is using the polymerase chain reaction (PCR; for DNA amplification) and reverse transcription-PCR (RT-PCR, for RNA) [1]. While PCR and RT-PCR assays have a theoretical detection limit of one genomic copy per reaction, the presence of certain compounds, enzymes, and ions in environmental water samples can inhibit the proper functioning of RT and PCR enzymes when these materials are present in nucleic acid samples used for molecular testing. Thus, for sensitive molecular detection of waterborne microbes without prior culture, nucleic acid (*i.e*., DNA and/or RNA) must be effectively extracted from microbial cells and separated from interfering substances.

Most microbes are susceptible to chemical and mechanical lysis, with chemical lysis typically being sufficient for efficient extraction of DNA and RNA from viruses and bacteria [2,3]. While vegetative bacteria are considered to be relatively easy to lyse, with simple boiling often being sufficient to achieve good DNA extraction and PCR detection [4], the effectiveness of lysis techniques can be greatly affected by the composition of bacterial cell walls. For example, Gram-positive bacteria have been shown to be more resistant to lysis than Gram-negative bacteria because of their rigid peptidoglycan cell wall structure [5]. Bacterial spores are more resistant to chemical lysis and physical disruption than vegetative bacteria, likely due to their tough, peptidoglycan-based spore cortex [6]. Protozoan parasite cysts and oocysts, especially *Cryptosporidium parvum* oocysts, are known be difficult to lyse using chemical and mechanical disruption techniques. The oocyst wall of *C. parvum* has been shown to be composed of two to three distinct layers that combine to protect the internal sporozoites [7].

Although both RNA and DNA can be extracted from microbes using many of the same techniques, RNA is generally less stable than DNA and is more susceptible to degradation if not properly protected and stored. RNA degrading enzymes (e.g., RNase) are especially of concern in environmental samples, and must be inactivated prior to, or in conjunction with, RNA extraction [8]. RNA and DNA differ in other properties (e.g., solubility and pKa) that can impact the effectiveness of separation and purification techniques [9,10]. While it is common to see commercial RNA- and DNA-specific extraction and purification products, methods for simultaneous extraction and recovery of DNA and RNA have been reported [3,11,12,13,14] and a variety of “total nucleic acid” extraction kits are commercially available.

Effective extraction of DNA and RNA from water samples can be problematic because of the high variability in water quality, which can be affected by season effects and weather events. Water can contain diverse substances, including soil, sediment, plant matter, and various dissolved inorganic and organic compounds. Plant matter contains phenolics, polysaccharides, and other substances that can interfere with nucleic acid extraction and amplification. In addition, one of the primary applications for molecular testing of water samples is monitoring water for the presence of pathogenic microbes and indicators of fecal contamination. These microbes are typically present in water at low concentrations, which makes it difficult to optimize extraction methods to achieve both high nucleic acid recovery and purity. However, optimization of nucleic acid extraction and purification procedures is critical for effective application to water samples, as a wide range of impurities, including dissolved organic matter, salts, detergents, or organic solvents can inhibit PCR and RT-PCR. Organic matter in water has been described as consisting of three general groups (humic acid, fulvic acid, and humin), with humic acid comprising the majority of organic matter in water and representing the greatest potential for RT and PCR inhibition [15,16]. Humic substances can be co-extracted with nucleic acid and inhibit nucleic acid polymerase enzymes, such as *Taq* polymerase [17,18,19]. Although environmental compounds can inhibit both RT and PCR, inhibitors are generally considered to have greater potential for inhibiting RT (and thus be a more significant issue for molecular detection of RNA targets than DNA targets) [15]. In addition, nucleases such as ribonucleases (RNases), are present in environmental samples and if not inactivated during nucleic acid extraction these enzymes can degrade extracted nucleic acid [20].

In this study, alternative lysis buffer components were investigated to develop a lysis buffer for extracting DNA and RNA from diverse waterborne microbes, including vegetative bacteria (*Salmonella*), bacterial spores of *Clostridium perfringens*, viruses (adenovirus type 40, norovirus [human GII.13 and murine]), and parasites (*C. parvum* and *Giardia duodenalis*).

## 2. Results

### 2.1. Evaluation of Reducing Agents

While reducing agents such as 2-mercaptoethanol and dithiothreitol have been reported in previous studies as being effective reducing agents in lysis buffers [21,22], there was concern that incorporation of these toxic and hazardous reagents could limit the widespread use of the final protocol by water laboratories. Experiments were therefore performed to compare less toxic reducing agents, sodium sulphite and dithioerythritol (DTE), to 2-mercaptoethanol as potential components of the lysis buffer. Both an environmental water control (*i.e.*, Utility A source water extracted without reducing agents in the buffer) and a reagent-grade water control were used. These controls enabled comparison of reducing agent-associated CT values with a best-case condition (reagent-grade water control) and worst-case condition (Utility A source water extracted without reducing agents). This combination of sodium sulphite (SS) and DTE was associated with lower average CT values than use of DTE or 2-mercaptoethanol alone (Table 1). Higher amounts of microbial nucleic acid were detected when using “DTE + SS” compared to the environmental water control, 2-mercaptoethanol, or DTE alone. CT values for the reagent-grade water control were significantly lower than the DTE + SS condition, but the overall difference in mean CT value was relatively small (29.1 for the control and 31.8 for DTE + SS). Use of 2-mercaptoethanol and DTE alone was not found to be associated with significantly different CT values. Based on these results, DTE (0.2% lysis buffer concentration) was incorporated into the lysis buffer along with 0.2% sodium sulphite.

**Table 1:** Effect of reducing agents in GITC-based lysis buffer for molecular detection of different enteric microbes in source water (CT values ± SD) *.

Microbe	DTE	2-mercapto	DTE + SS	Source water Control	Reagent-Grade Water Control
AdV40	29.6 ± 0.2	29.5 ± 0.5	27.0 ± 1.3	29.7 ± 0.3	24.8 ± 0.2
T4	36.4 ± 3.0	35.4 ± 2.8	30.0 ± 7.9	35.7 ± 2.5	28.0 ± 0.4
HuNoV	28.0 ± 2.0	27.9 ± 2.2	28.2 ± 2.3	28.3 ± 2.3	25.9 ± 0.9
MNV	34.2 ± 3.2	33.2 ± 3.2	33.2 ± 3.0	33.5 ± 3.4	31.6 ± 0.7
*C. perfringens* spores	32.6 ± 1.2	32.4 ± 1.7	30.6 ± 2.0	32.6 ± 2.7	26.8 ± 0.8
*S.* Typhimurium	38.2 ± 4.2	38.2 ± 4.9	33.6 ± 2.8	40.5 ± 4.3	32.1 ± 1.3
*C. parvum*	39.6 ± 3.1	39.0 ± 3.5	34.5 ± 1.6	38.7 ± 3.7	31.9 ± 0.3
*G. duodenalis*	34.6 ± 1.1	34.9 ± 0.4	33.7 ± 0.2	34.7 ± 3.9	32.0 ± 1.0

* N = 3 for all microbes.

### 2.2. Bead Beating

At this point in the study, the lysis buffer contained the following components as a 2X buffer:

- 4.5 M GITC dissolved in Tris(10 mM)-EDTA(1 mM) (TE) buffer (pH 8.0)
- polyadenylic acid [poly(A)] (17.6 μg/mL)
- 0.14 M sodium acetate (NaOAc)
- 0.24 M NaCl
- 0.4% sodium sulphite
- 0.2% dithioerythritol (DTE)
- 0.02% Sodium dodecyl sulfate (SDS)
- 0.4% Tween 20

Alternative types and combinations of different beads were investigated to determine which were effective for improving DNA and RNA extraction from the suite of study microbes. No significant differences were observed between glass and zirconium oxide (ZrO_x_) beads, either alone or in combination, at different bead sizes (0.1, 0.2 and 0.5 mm) (data not shown). However, a combination of two ZrO_x_ bead sizes provided greater improvements in analytical sensitivity for *S.* Typhimurium, *C. perfringens* spores and *C. parvum* than the slight improvements in analytical sensitivity associated with a combination of two glass bead sizes (for HuNoV and *G. duodenalis*) (Table 2). The combination of 0.2 and 0.5 mm ZrO_x_ beads was selected for use as part of the final nucleic acid extraction protocol.

**Table 2:** Effect of bead beating on the performance of GITC-based lysis buffer for the detection of enteric microbes *.

Experiment Condition	*S.* Typhimurium	*C. perfringens* spores	HuNoV	*C. parvum*	*G. duodenalis*
No beads	32.2	29.0	26.5	33.6	36.1
0.1 + 0.5 mm Glass	35.6	29.8	26.4	35.6	33.2
0.2 + 0.5 mm ZrO_x_	33.0	27.8	28.0	32.8	34.2

* N = 2 for each experiment condition.

### 2.3. Proteinase K Digestion

Next we evaluated the effect of Proteinase K digestion in conjunction with different lysis buffer compositions on the detection of the panel of enteric microbes. When no bead beating was performed, the SDS and SDS-Proteinase K conditions resulted in the lowest overall mean CT values, with the exception of T4 phage (Table 3). When bead beating was performed in conjunction with each lysis buffer condition all microbes could be detected consistently. The results for the SDS-Tween 20-Proteinase K lysis buffer resulted in significantly higher yield of detectable nucleic acid than the other buffer compositions.

**Table 3:** Average CT values from duplicate experiments investigating alternative final lysis buffer compositions.

Experiment Condition	*Salmonella*	*C. perfringens* spores	AdV40	HuNoV	MNV	T4 phage	*C. parvum*
Alternative Final Lysis Buffer Components (no bead beating)							
SDS	36.5 ^b^	26.5	24.8	37.9	31.2	39.6	36.2 ^b^
SDS-Proteinase K	37.0	25.7	22.8	38.6	29.9	36.9	40.4 ^b^
SDS-Tween 20	Neg ^a^	25.8	24.0	37.2	33.6	28.6	Neg
SDS-Tween 20-Proteinase K	39.1 ^b^	25.8	23.8	41.4	33.0	39.3	37.4 ^b^
Alternative Final Lysis Buffer Components (with bead beating)							
SDS	35.1	27.4	25.8	41.8	31.8	31.0	36.1
SDS-Proteinase K	34.6	27.2	26.1	33.6	31.3	30.6	36.2
SDS-Tween 20	34.6	26.8	25.5	36.4	33.2	30.0	34.6
SDS-Tween 20-Proteinase K	33.2	26.0	24.2	33.9	31.2	29.6	33.2

^a^ Neg = both reactions were negative; ^b^ One of the two reactions was negative, so CT value for one positive reaction reported.

### 2.4. Nucleic Acid Purification and PCR Facilitators

Because concentrated water samples contain diverse constituents that can inhibit PCR and RT-PCR, experiments were performed to evaluate nucleic acid purification techniques (polyvinylpolypyrrolidone [PVPP] spin column or Sephadex G-100 spin column *versus* a silica column-only control) and PCR facilitators (non-acetylated bovine serum albumin [BSA], betaine, GC-RICH [Roche Molecular Diagnostics, Pleasanton, CA], and bacteriophage T4, gene 32 protein [gp32] added to PCR and RT-PCR mastermix). Overall, no significant differences in performance were identified for either of the nucleic acid purification spin columns *versus* the control (Table 4). None of the purification techniques were associated with appreciably lower CT values (*versus* the control) for AdV40 or *C. perfringens* spores. The PVPP spin column was associated with slightly lower average CT values for HuNoV and *Giardia* (2.3 and 1.4 CT values, respectively). However, for *C. parvum* addition of PVPP resulted in an average of 1.4 CT values higher than the control. The use of a Sephadex G-100 column alone did not result in lower CT values with the possible exception of *Salmonella*, for which two of three reactions were positive *versus* the control (for which all three replicate assays were negative). While no significant differences were observed, the use of a PVPP column was associated with lower CT values for HuNoV *versus* G-100 and the control, so use of a PVPP spin column was incorporated into the final UNEX extraction procedure.

**Table 4:** Average ± standard deviation CT values for three replicate experiments investigating polyvinylpolypyrrolidone (PVPP) and G-100 inhibitor removal columns.

Analyte	PVPP	G-100	Control
AdV40	24.1 ± 0.4	24.3 ± 0.4	23.4 ± 0.3
HuNoV	28.6 ± 0.5	30.2 ± 0.5	30.9 ± 0.2
MNV	36.7 ± 1.5	36.3 ± 0.2	36.6 ± 1.1
*C. perfringens* spores	28.5 ^d^	28.2 ± 0.6	27.9 ± 0.4
*Salmonella*	37.9 ^c^	37.5 ^b^	Neg ^a^
*C. parvum*	33.7 ± 2.4	32.1 ± 0.1	32.3 ± 0.8
*G. duodenoalis*	34.2 ± 0.8	34.8 ± 1.1	35.6 ± 0.3

^a^ Neg = all three reactions were negative; ^b^ CT values averaged for two of three reactions (one reaction was negative); ^c^ CT value for one positive reaction (two of three reactions were negative); average of two reactions (third reaction failed and could not be repeated); ^d^ average of two reactions (third reaction failed and could not be repeated).

The results of the facilitator experiments indicated that betaine and GC-RICH were not associated with lower average CT values *versus* the control for *Salmonella*, *C. perfringens* spores, or MNV, but negatively affected the detection of *C. parvum*, T4 bacteriophage, and HuNoV (Table 5). Inclusion of gp32 in the RT-PCR reaction mix was associated with lower average CT values for HuNoV and MNV. For the other microbes, no significant differences in average CT values were found. Non-acetylated BSA was also associated with lower average CT values for HuNoV and MNV, but the differences in average CT values were not as large as for gp32. While no overall significant differences were observed, non-acetylated BSA and gp32 were included as PCR facilitators to reduce the potential impact of RT-PCR inhibitors on the detection of microbes in water samples.

**Table 5:** Average CT values for two replicate experiments investigating PCR and RT-PCR facilitators.

Analyte	BSA	Betaine	GC-RICH	gp32	Control
T4	37.6	41.6 ^b^	37.2 ^b^	36.6	35.4
HuNoV	34.8	35.3 ^b^	Neg	28.0	36.1
MNV	40.3	37.8 ^b^	Neg	34.9	40.3 ^b^
*C. perfringens* spores	33.0	31.8 ^b^	35.4	30.2	30.0
*Salmonella*	33.2	34.0	33.8	34.0	33.2
*C. parvum*	36.0	40.7 ^b^	Neg ^a^	36.9	35.2

^a^ Neg = both reactions were negative; ^b^ One of the two reactions was negative, so CT value for one positive reaction reported.

### 2.5. Final Method Evaluation versus Commercial Kits and with Panel of Water Concentrates

**Figure 1:**
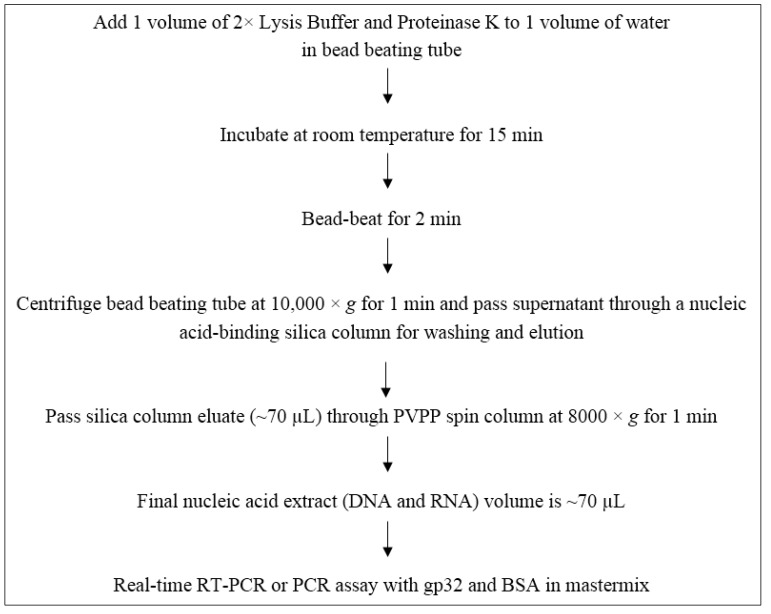
Flow diagram of optimized UNEX buffer protocol for the molecular detection of enteric microbes in water samples.

The results of the technique evaluation experiments led to the development of the nucleic acid extraction, purification and analysis method identified in Figure 1.

The procedure shown in Figure 1, including pre-treatment with proteinase k, lysis of the microbes with the UNEX buffer, and inclusion of PCR facilitators, was then compared against two commercial DNA extraction kits (UltraClean Soil DNA Kit [MO BIO Laboratories] and FastDNA Spin Kit for Soil [MP Biomedicals]) commonly used for extraction of environmental samples. The only microbes for which the UNEX buffer protocol performed poorer was for MNV (*versus* the UltraClean kit) and *Salmonella* (*versus* the FastDNA kit) (Table 6). These data provided further evidence that the UNEX buffer-based extraction protocol enabled effective detection of enteric microbes in concentrated water samples compared to commercial kits.

**Table 6:** Comparison of the UNEX buffer protocol with two commercial kits *.

Analyte	UNEX Buffer Method	UltraClean Soil DNA Kit	FastDNA Spin Kit for Soil
HuNoV	21.9 ± 0.4	21.6 ± 0.6	28.2 ± 1.5
MNV	26.8 ± 0.3	23.4 ± 0.2	31.1 ± 0.7
*C. parvum*	31.0 ± 0.6	40.1 ± 4.2	33.7 ± 1.2
*Salmonella*	31.9 ± 0.4	35.9 ± 0.7	27.9 ± 0.3
*C. perfringens* spores	25.4 ± 0.7	33.3 ± 0.6	30.4 ± 0.6

* N = 3 for each analyte.

In experiments performed using a panel of 10 water samples (plus reagent-grade water control), the UNEX buffer sample preparation protocol was generally associated with real-time PCR and RT-PCR CT values that were similar (*i.e.*, within 3 CT values) of the CT values produced for the control water samples (Table 7). The most challenging water samples to analyze were from Utility A, for which average PCR or RT-PCR CT values were generally > 3 CT values *versus* the reagent-grade water control. These results may have been due to lower water quality, as suggested by turbidity and organic carbon data (Appendix Table A1). For the other eight water samples (excluding Utility A samples), the UNEX buffer method yielded average CT values that were within 3 CT values of the Control for each of the DNA analytes, with the exception of four analytes (*Salmonella* and *C. parvum* data for Utility C source water and AdV40 data for Utility C and D source waters).

**Table 7:** Average CT values (± std. dev.) for extracted nucleic acid from panel of seeded water concentrates collected from five water utilities *.

Utility/Sample Type	AdV40	HuNoV	MNV	T4	*Sal.*	*C. perf.*	*C. parvum*	*Giardia*
Control Water	27.2 (0.1)	24.8 (0.6)	29.1 (0.1)	30.6 (0.2)	30.8 (0.1)	31.3 (0.0)	37.9 (0.9)	33.6 (0.2)
Utility A/Source	34.8 (0.4)	34.4 (1.3)	33.2 (0.2)	38.4 (1.5)	34.8 (0.1)	38.5 (1.6)	42.1, Neg	38.1 (0.7)
Utility A/ Finished	36.0 (0.8)	35.1 (1.4)	34.0 (0.5)	38.7 (0.4)	35.4 (0.4)	38.3 (0.5)	Neg	39.0 (0.6)
Utility B/Source	27.3 (0.1)	28.0 (0.7)	28.0 (0.7)	30.5 (1.1)	28.4 (0.1)	30.3 (0.6)	38.2 (1.2)	33.4 (0.5)
Utility B/ Finished	26.9 (0.2)	26.5 (0.9)	26.5 (0.9)	30.9 (1.6)	29.4 (0.1)	30.4 (0.3)	38.0 (0.5)	32.7 (0.9)
Utility C/Source	31.4 (0.3)	28.8 (1.0)	28.8 (1.0)	32.1 (0.2)	33.9 (0.5)	33.7 (0.3)	39.8, Neg	35.3 (0.1)
Utility C/ Finished	27.2 (0.2)	32.4 (1.1)	32.4 (1.1)	30.4 (0.4)	30.8 (0.2)	29.6 (0.3)	35.9 (0.5)	32.8 (0.4)
Utility D/Source	30.5 (0.1)	30.3 (0.8)	33.2 (0.4)	32.7 (0.5)	33.3 (0.5)	33.1 (0.4)	40.0 (2.7)	34.4 (0.6)
Utility D/Pre-Finished	29.6 (0.2)	26.4 (1.4)	29.3 (0.1)	31.0 (0.5)	30.8 (0.1)	31.7 (0.4)	38.6 (2.1)	34.0 (0.3)
Utility E/Source	26.5 (0.4)	24.8 (1.3)	28.4 (0.3)	29.2 (0.4)	29.1 (0.1)	30.1 (0.3)	35.5 (0.6)	33.0 (0.5)
Utility E/ Finished	26.8 (0.3)	33.0 (2.1)	36.5 (1.1)	29.9 (0.1)	30.2 (0.6)	29.3 (0.1)	36.0 (0.8)	33.7 (0.1)

* N = 3 for each utility/sample type.

Results for the RT-PCR analytes (HuNoV and MNV) demonstrated the challenge of removing RT inhibitors from environmental water samples. Only one source water (Utility E), one finished water (Utility B) and the Utility D pre-finished water were associated with average CT values for HuNoV that were within 3 CT values of the Control. Results were better for MNV, for which five of the ten water samples were associated with average CT values that were within 3 CT values of the Control. However, significant RT-PCR inhibition (as much as 10 CT values) was observed for HuNoV assays for many of the water samples (particularly, Utility A samples, Utility C finished water, and Utility E finished water) suggesting that for this important public health pathogen additional purification may be needed for sensitive detection in concentrated large-volume water samples.

## 3. Discussion

The results of this study indicate that the lysis buffer developed specifically for extraction and recovery of microbial DNA and RNA from water samples (*i.e*., “UNEX buffer”) was effective for improving real-time PCR and RT-PCR analyses for a diverse set of microbes (including viruses, vegetative bacteria, bacterial spores, and protozoan parasite (oo)cysts). The UNEX buffer contains components that have been found to be effective for the extraction of enteric microbes including GITC, SDS, Tween 20, and Proteinase K [21,23,24]. When Tween 20 and SDS were evaluated in combination and in conjunction with Proteinase K, improved results were obtained for all microbes when bead beating was performed. Proteinase K is a widely used lytic enzyme, especially for protozoan parasite (oo)cyst digestion [7]. The addition of DTE and sodium sulphite to the lysis buffer, as less toxic alternatives to more commonly used reducing agents (e.g., DTT, 2-mercaptoethanol), enabled significantly more sensitive detection *versus* environmental water controls (*i.e.*, were significantly effective for reducing PCR inhibition). When used in conjunction with bead beating, the UNEX buffer was found to be effective in extracting DNA and RNA from diverse microbes in large-volume water concentrates. The bead beating procedure was simple, although non-commercial beads (Yttrium-stabilized zirconium oxide beads) yielded better nucleic acid extraction results compared to commercially available glass beads. While the zirconium oxide beads were incorporated into the final water sample preparation protocol, our data suggest that users of this protocol could replace the zirconium oxide beads with equivalent-sized glass beads with relatively little reduction in method performance.

Experiments performed with UF-concentrated water samples enabled refinement of the lysis buffer conditions (e.g*.*, selection of reducing agents), as well as identification of the PVPP spin column as an effective component for removing PCR and RT-PCR inhibitors. The final water sample preparation procedure (Figure 1) was simple, could be completed within 45 min, and was found to provide similar or better performance *versus* commercially available nucleic acid extraction kits. The water panel testing data (Table 7) showed that the UNEX buffer and sample preparation procedure could effectively extract and recover DNA and RNA from concentrated large-volume water samples. Still, the testing data for some of the water samples (e.g., from Utility A) indicate that further improvement of the method is warranted, especially as new PCR inhibitor removal techniques become available. As expected, removal of RT-PCR inhibitors was the biggest challenge for developing the sample preparation procedure. However, while significant RT-PCR and PCR inhibition was observed for some of the water samples, it should be noted that for each water sample (reflecting 40 L for source water and 100 L for finished water), approximately 0.9% of each sample was tested by PCR and RT-PCR. So it was not surprising to observe substantial PCR and RT-PCR inhibition, especially for source water samples having turbidity as high as 9 NTU, when each reaction is testing ~360 mL of an original 40-L water sample.

This study has several limitations. First, comparisons in the performance of alternative nucleic acid extraction and purification procedures were performed using CT values. This approach was chosen for analytical efficiency, but did not allow for direct comparative analyses of detection limits associated with the alternative procedures (which would have required performing additional experiments using different microbial seed levels). The relative performance data reported in this study were for a suite of eight microbes and may not reflect the performance of the extraction and purification procedures for other microbes. Second, ultrafiltration and centrifugation were used as a model sample processing procedure to produce concentrated water samples for this study. This approach produced final samples having “worse-case” sample quality because ultrafiltration captures all particles in a water sample and long-chain organics (e.g., humic acids) having a molecular weight higher than the molecular weight cut-off of the ultrafilters (e.g., 30,000 daltons) [25]. The relative PCR and RT-PCR performance data reported for the UNEX buffer-based protocol (Table 7) may have been closer to the control if smaller volume water samples were used (*i.e.*, smaller than 100 L finished water, 40 L source water) and if other filtration techniques (e.g., microfilters instead of ultrafilters) were used. Third, the data reported here were collected as part of a larger study in which many other nucleic acid extraction and purification procedures were evaluated [26]. Because of the complexity and time constraints associated with the larger study, only three replicate experiments were generally performed when evaluating the different nucleic acid extraction and purification procedures. Fourth, when comparing the UNEX buffer-based protocol with commercial kits, we followed vendor guidance for the amount of sample to extract and final nucleic acid extract volumes for the kits, which differed slightly from the volumes used for the UNEX protocol. These differences were not substantial, but may have affected resulting CT values for the different extraction methods.

## 4. Materials and Methods

### 4.1. Microbe Sources

*Cryptosporidium parvum* oocysts and *Giardia duodenalis* cysts were obtained from Dr. Becky Hoffman’s laboratory at the Wisconsin State Laboratory of Hygiene. Microcentrifuge tubes containing 100 μL TE Buffer were used to receive sorted *C. parvum* and *G. duodenalis* (oo)cysts which were quantified by flow cytometry to contain 200 ± 2.1 *C. parvum* oocysts and 200 ± 2.3 *G. duodenalis* cysts. *Salmonella enterica* subsp. *enterica* serovar Typhimurium “BioBalls” containing 250 cfu of were acquired through a special order from BTF Precise Microbiology (Australia). *Clostridium perfringens* spores were also obtained as BioBalls from BTF. Each *C. perfringens* BioBall contained 10,000 cfu. One BioBall was used to seed each water sample that was extracted to investigate a particular lysis buffer condition. A standard stock of adenovirus 40 (AdV40, Dugan strain, [27]) was prepared and partially purified by chloroform extraction [27] and the number of virus particles were quantified as 5.1 × 10^9^ particles/mL. In order to minimize potential freeze-thaw damage of the viruses, aliquots of 10,000 virus particles/μL were frozen at −70 °C. A virus stock of murine norovirus (MNV) was prepared in RAW cells by standard techniques [28] and contained 7.1 × 10^8^ particles/mL as determined. As done for AdV40, dilutions of the MNV stock were made to achieve a final concentration of 10,000 particles/μL. Electron microscopy analysis was also performed on the frozen and thawed stocks to demonstrate that virus particles remained intact. Initially, 100 tubes (50 μL/tube) were aliquoted and frozen at −70 °C. A human norovirus (GII.13; HuNoV) positive stool sample was obtained from a cruise outbreak reported to CDC. The extracted norovirus stock from this stool sample was determined to have a concentration of 1.5 × 10^9^ particles/mL by electron microscopy. T4 bacteriophage 4.2 × 10^7^ pfu/mL were purchased from Attostar LLC as a DNA extraction and PCR inhibition control product. For lysis buffer development experiments, we made T4 bacteriophage aliquots of 42,000 pfu/μL.

### 4.2. Microbe Seeding

*C. parvum* and *G. duodenalis* (oo)cysts, were centrifuged to collect all material in the tip of the tube. One *C. perfringens* and one *Salmonella* BioBall were then added and the tube was then centrifuged at 10,000 × *g* for 5 min and 97 μL of TE buffer was removed. Fresh TE buffer (97 μL) was added to the microfuge tube and spun at 10,000 × *g* for 5 min. The supernatant (~97 μL) was removed and 5 μL of AdV40, 5 μL of MNV, and 5 μL of T4 bacteriophage was added. The final volume was made up to 250–500 μL with nuclease-free water or utility water, depending on the goal of the experiment. The input microbial levels for all experiments were: 50,000 particles of AdV40 and MNV, 75,000 particles of GII.13 norovirus, 250 cfu *Salmonella*, 10,000 cfu *C. perfringens* spores, 200 *C. parvum* oocysts, 200 *G. duodenalis* cysts, and 210,000 PFU of T4 bacteriophages.

### 4.3. Water Samples

Nuclease-free, reagent-grade water (Ambion, Foster City, CA) was used for method development experiments, unless otherwise noted. Water samples were obtained from each of five US water utilities (Utilities A-E). Each utility supplied at least two 40-L samples of source water (*i.e.*, raw water influent to the utility water treatment facility) and two 100-L samples of finished water from the water treatment facility. Each water sample was concentrated using tangential flow, hollow-fiber ultrafiltration using Fresenius F200NR or Baxter Exeltra Plus 210 ultrafilters as described previously [29]. However, no water sample amendments, filter blocking, or filter backflushing was performed when concentrating the water samples. Ultrafilter retentate samples were further concentrated by centrifuging the entire retentate sample at 4000 × *g* for 30 min in a 500 mL conical tube. The pelleted materials were resuspended with the remaining water sample and the final concentrate transferred to a 15 mL conical tube and stored at 4 °C. Final resuspended pellet volumes were 4.5 ± 2.2 mL for source water samples and 3.9 ± 0.5 mL for finished water samples. Packed debris pellet volumes were not measured.

### 4.4. Water Quality Testing

Water utility water samples were analyzed for the following suite of water quality parameters: pH, turbidity, total organic carbon (TOC), dissolved organic carbon (DOC), specific conductance, and alkalinity. Sample pH was measured with an Accumet^®^ Research AR25 pH/mV/°C/ISE Meter (Thermo Fisher, Waltham, MA, USA). Turbidity was measured using a Model 2100N Laboratory Turbidimeter (Hach Company, Loveland, CO, USA). TOC and DOC were both measured using the Hach Low Range Total Organic Carbon (TOC) Reagent Set and the Hach DR/2400 Portable Spectrophotometer. To process a sample for DOC, an Ahlstrom 0.7 μm pre-baked borosilicate microfiber disc filter (Environmental Express, Charleston, SC, USA) was conditioned by filtering 300 mL deionized (DI) water. Then 60 mL of the sample was passed through the filter and the last 10 mL was retained for analysis. Specific conductance was measured with an Oakton CON 100 Conductivity/°C meter. Alkalinity was determined using a Hach Alkalinity Test Kit, Model AL-DT, Digital Titrator.

### 4.5. Lysis Buffer Components

Guanidinium isothiocyanate was chosen as the base lysis buffer, along with common salts (sodium chloride [NaCl] and sodium acetate [NaOAc]) and carrier RNA, based on prior research [30]. 4.5 M GITC (Roche Diagnostics, Indianapolis, IN) was made in TE buffer (pH 8.0) and dissolved at 50 °C for 30 min. Carrier nucleic acid, poly(A) (Sigma-Aldrich, St. Louis, MO, USA), was added to the base lysis buffer at a concentration of 17.6 μg/mL to counteract potential inefficiencies associated with extracting low levels of nucleic acid from some microbes. All lysis buffer development experiments were performed by adding lysis buffer solution to a seeded water sample at a ratio of 1:1, and vortexing to mix the buffer and water sample.

Alternative salt, surfactant and reducing agent additives to the guanidinium-based lysis buffer were prepared using molecular-grade water, and appropriate stock volumes added to the guanidinium-based lysis buffer to achieve target concentrations. Commonly used reducing agents in lysis buffers include 2-mercaptoethanol and DTT, but there are health and safety concerns with handling these toxic chemicals, so alternatives were investigated. Baranwal *et al.* and Singh *et al.* reported using the non-toxic reagent, sodium sulphite, as a reducing agent for protecting DNA and RNA extracted from plant matter [31,32]. In addition, DTE, a reagent that can break disulfide bonds of compounds that can inhibit RT and PCR enzymes, was investigated as a less toxic alternative to DTT. The following final concentrations of reducing agents were identified from previous studies and investigated in the present study: 1% 2-mercaptoethanol, 0.2% and 0.1% sodium sulphite, and 0.1% DTE. Triplicate experiments were performed using 250-μL aliquots of concentrated Utility A source water.

When the lytic enzyme, Proteinase K (Qiagen, Valencia, CA), was studied, 125 μL of the enzyme (20 mg/mL stock concentration) was added to a volume of 1 mL of the lysis buffer-water sample mixture. After addition, the sample was allowed to stand at room temperature for 15 min before processing using a silica column (HiBind RNA Minicolumn, Omega Bio-Tek, Norcross, GA), with or without prior bead beating. The effect of Proteinase K was evaluated in duplicate experiments using 350 μL aliquots of deionized water.

### 4.6. Bead Beating

Bead beating was performed using glass beads (Sigma-Aldrich, St. Louis, MO) and zirconium oxide, ZrO_x_ (Y_2_O_3_ stabilized) beads (Union Process, Akron, OH). The glass beads studied were 425–600 μm (Sigma # G-8772) and ≤ 106 μm (Sigma # G-4649) in size. The zirconium oxide beads studied were 1 mm (Union Process # 0087-01), 0.5 mm (Union Process # 0087-05), and 0.2 mm (Union Process # 0087-02). The zirconium oxide (ZrO_x_) beads were reported to be Y_2_O_3_- [Yttrium (III) oxide, or “Yttria”] stabilized and “high purity” (95%) by the vendor. The glass beads from Sigma were acid washed by the company, but the beads obtained from Union Process were raw materials. These beads were washed three times with deionized water, then washed once with 0.1N HCl, followed by three DI water rinses to remove the acid. After washing, the beads were dried in an oven at 200 °C for 30 min.

For each bead condition studied, a microcentrifuge tube “capful” of each bead type (or multiple bead types) was added to an nuclease-free, 2.0 mL bead beating tube (Biostor Vials, Cat No. BC20NA-PS, National Scientific Supply, Claremont, CA). A capful of beads weighed ~200 mg. The seeded water sample in lysis buffer (500–1000 μL, depending on the experiment) was then added to the bead beating tube. The tube was then loaded (along with tubes for the other conditions studied in the experiment) into a Mini-Bead-Beater-8 instrument (BioSpec Products, Inc., Bartlesville, OK, USA). After bead beating for 2 min, each tube was taken out of the bead beating instrument, centrifuged at 10,000 x *g* for 1 min, and the supernatant transferred to a silica column for processing. If more than 700 μL of supernatant was present in a tube, then the additional sample was passed through the silica column as a second sample processing step. Bead comparison experiments were performed in duplicate using 350 μL aliquots of deionized water.

### 4.7. Nucleic Acid Separation and Purification

Silica columns (Omega BioTek, Norcross, GA) were used to simultaneously capture and concentrate RNA and DNA for all experiments. The 500–1000 μL volumes (lysis buffer + seeded sample) were placed in a silica column and centrifuged for 1 min at 10,000 × *g*. The silica column was washed with 500 μL of 100% ethanol and centrifuged for 1 min at 10,000 × *g*, followed by washing with 500 μL of 75% ethanol and centrifuging for 1 min at 10,000 × *g*. A final dry spin for 1 min at 10,000 × *g* was performed to remove residual ethanol from the silica column. When a silica column was the only nucleic acid separation/purification technique used in a protocol, the column was eluted with 70 μL of TE buffer for 1 min at 10,000 × *g*. A polyvinylpolypyrrolidone (PVPP) spin column (Spin-IV-HRC, Zymo Research, Irvine, CA) was centrifuged at 8000 × *g* for 1 min when used in conjunction with a silica column. Sephadex G-100 (Epicentre Biotechnologies, Madison, WI) columns were produced by pipetting Sephadex G-100 into filter spin tubes provided by the vendor. Triplicate experiments comparing PVPP and Sephadex G-100 columns were performed using 200 μL aliquots of concentrated Utility A source water.

Non-acetylated BSA (Sigma-Aldrich, St. Louis, MO), betaine (Sigma-Aldrich, St. Louis, MO), gp32 (New England Biolabs, Ipswich, MA), and GC-RICH (Roche Molecular Diagnostics, Pleasanton, CA, USA) were studied to determine their potential effectiveness to facilitate PCR and RT-PCR in the presence of inhibitors from extracted water samples. The addition of BSA and gp32 in PCR reaction mixes have shown to reduce PCR and RT-PCR inhibition in water and other environmental samples [33,34,35]. Betaine has been reported to be effective for facilitating the amplification of GC-RICH DNA sequences [36]. The PCR facilitators were investigated at concentrations of 400 ng/μL (non-acetylated BSA), 25 ng/μL (gp32), and 1M (betaine) while GC-RICH was used per manufacturer’s protocol. For these duplicate experiments, 250 μL volumes of Utility A UF-concentrated source water were seeded with the suite of study microbes and nucleic acid extracted using bead beating (with lysis buffer). Extracted nucleic acid was purified using a silica column and a PVPP column.

### 4.8. PCR and RT-PCR Conditions

Real-time PCR and RT-PCR assays were performed in separate thermal cycler runs. All PCR and RT-PCR assays (Table 8) were performed in the same thermal cycler to enable direct comparison of the effect of different buffer components or PCR facilitators on crossing threshold (CT) values for each microbe. All method development PCR assays were performed using Qiagen QuantiTect Probe PCR Kits on a Bio-Rad iQ4 Real-Time PCR System using the following thermal cycling conditions: denaturation at 95 °C for 15 min, followed by 45 cycles of denaturation at 95 °C for 10 s, annealing at 55 °C for 30 s, and extension at 72 °C for 20 s. Similarly, all method development RT-PCR assays were performed using QuantiTect Probe RT-PCR Kits on a Bio-Rad iQ4 Real-Time PCR System using the following reverse transcription and thermal cycling conditions: RT at 50 °C for 30 min, denaturation at 95 °C for 15 min, followed by 45 cycles of denaturation at 95 °C for 10 s, annealing at 55 °C for 30 s, and extension at 72 °C for 20 s.

Eight real-time PCR and RT-PCR assays were performed at various times during this project to generate CT value data for the following analytes: AdV40, norovirus GII, MNV, T4 bacteriophage, *Salmonella*, *C. perfringens*, *C. parvum*, and *G. duodenalis*. Positive and negative controls were performed for each assay and thermal cycler run. DNA and RNA from stocks of each study microbe (except norovirus GII) were used as positive controls. A GII RNA transcript was used as a positive control for the norovirus GII RT-PCR assay.

**Table 8:** Oligonucleotide primers and probes sequences used in this study.

Microbial Target	DNA sequence (5′-3′)	Reference
pan-Adenovirus	Forward primer, JTVXF, 5′-GGACGCCTCGGAGTACCTGAG-3′	[37]
	Reverse primer, JTVXR, 5′-ACIGTGGGGTTTCTGAACTTGTT-3′	
	Probe, JTV, XP, 5′-FAM-CTGGTGCAGTTCGCCCGTGCCA-BHQ-3′	
Norovirus, GII	Forward primer, JJV2F, 5′-CAAGAGTCAATGTTTAGGTGGATGAG-3′	[38]
	Reverse primer, COG2R, 5′-TCGACGCCATCTTCATTCACA-3′	
	Probe, RING2-TP, 5′-FAM-TGG GAG GGC GAT CGC AAT CT-BHQ-3′	
Murine norovirus	Forward primer, G54763F, 5′-TGATCGTGCCAGCATCGA-3′	[39]
	Reverse primer, G54863R, 5′-GTTGGGAGGGTCTCTGAGCAT-3′	
	Probe, G54808, 5′-FAM-CTACCCACCAGAACCCCTTTGAGACTC-BHQ-3′	
T4 bacteriophage	Forward primer, T4F, 5′-AAGCGAAAGAAGTCGGTGAA-3′	[40]
	Reverse primer, T4R, 5′-CGCTGTCATAGCAGCTTCAG-3′	
	Probe, T4P, 5′-FAM-CCACGGAAATTTCTTCATCTTCCTCTGGCCGTGG-BHQ-3′	
*Salmonella* sp.	Forward primer, 5′-GCCTTTCTCCATCGTCCTGA-3′	[25]
	Reverse primer, 5′-TGGTGTTATCTGCCTGACC-3′	
	Probe, 5′-FAM-TGCGATCCGAAAGTGGCG-BHQ-3′	
*C. perfringens*	Forward primer, 5′-CACAAGTAGCGGAGCATGTG-3′	[25]
	Reverse primer, 5′-CCCCGAAGGGATTTCCTCGATT-3′	
	Probe, 5′-FAM-AACCTTACCTACACTTGACATCCCTTGC-BHQ-3′	
*Cryptosporidium*	Forward primer, JVAF, 5′-ATGACGGGTAACGGGGAAT-3′	[41]
	Reverse primer, JVAR, 5′-CCAATTACAAAACCAAAAAGTCC-3′	
	Probe, JVAP18S, 5′-FAM-CGCGCCTGCTGCCTTCCTTAGATG-BHQ-3′	
*Giardia*	Forward primer, JVGIAF, 5′-ATCCGGTCGATCCTGCCG-3′	This study
	Reverse primer, JVGIAR, 5′-GGGGTGCAACCGTTGTCCT-3′	
	Probe, JVGIAP, 5′-FAM-CGGCGGACGGCTCAGGAC-BHQ-3′	

### 4.9. Final Method Evaluation *versus* Commercial Kits and with Panel of Water Concentrates

Two commercial nucleic acid extraction kits (UltraClean Microbial DNA Isolation Kit [MO BIO Laboratories, Carlsbad, CA] and FastDNA Spin Kit for Soil [MP Biomedicals, Santa Ana, CA]) were compared with the UNEX buffer sample preparation method as developed in this study. These kits were selected based on a review of the literature and vendor reports. For each method comparison experiment, a 3.5-mL source water UF concentrate sample from Utility A was seeded at the following microbe levels (per 500 μL volume of concentrate sample): AdV40 (50,000 particles), GII.13 norovirus (75,000 particles), MNV (50,000 particles), T4 bacteriophage (210,000 pfu), *Salmonella* Typhimurium (250 cfu), *C. perfringens* spores (10,000 cfu), *C. parvum* (200 oocysts), and *G. intestinalis* (200 cysts). The seeded water sample was then split to provide the starting sample volume for extraction, as directed by the vendor protocol for each kit. The following starting volumes were used for each kit/method: UNEX buffer (500 μL), FastDNA (200 μL), and UltraClean (300 μL). Final extract volumes were 70 μL for the UNEX method and FastDNA kit, and 50 μL for the UltraClean kit. Each kit/method was tested three times and all extract samples assayed in the same thermal cycler run (using 5 μL template per 20 μL reaction volume).

Source and finished water samples were received from each of the five participating water utilities. Each sample (40 L for source water, 100 L for finished water) was concentrated using ultrafiltration and then centrifuged to produce resuspended pellet volumes of ~4 mL. Three, 500-μL aliquots from each pellet sample were seeded with the same microbes and seed levels described previously for the kit/method comparison experiment. A non-seeded, 500-μL aliquot of each sample was also retained for background testing. The seeded and non-seeded samples were then extracted and purified using the UNEX buffer-based extraction protocol, and 5 μL of template assayed per 20-μL reaction volume in order to obtain the lowest detection level feasible for a 20-μL reaction. None of the study microbes were detected in the non-seeded water samples. A nuclease-free water control was also seeded and extracted. CT values from the water sample panel were then compared to CT values for the nuclease-free water control to evaluate the presence of PCR and RT-PCR inhibitors in the water sample extracts.

### 4.10. Data Analysis

CT values were used as the primary metric for evaluating the performance of the efficiency of the extraction buffer for each individual microbe seeded in water samples. Differences in CT values between methods were used as quantitative measures of the differences in effectiveness of alternative nucleic acid extraction methods. For a set of replicate experiments, the mean CT value and standard deviation were calculated for each method to evaluate performance differences. The non-parametric Friedman test (modified to allow multiple observations per experimental unit) was used to investigate differences in CT values for each microbial analyte. The test was performed utilizing SAS Proc GLM on the ranked data. Apriori differences among analytes were determined using either the associated LSMeans test for limited apriori comparisons or Tukey’s HSD test when multiple comparisons require that an experiment wide error rate of 0.05 be maintained. SAS version 9.1 was used for all Friedman test statistical analyses. In addition, Student’s t test was used for comparing CT values between two water samples (e.g., utility water sample and matrix control) for a single analyte (e.g., *Salmonella*). Statistical comparisons of CT values were only performed for samples tested in the same PCR runs (*i.e.*, on the same PCR plates). The alpha level was set at 0.05 for all statistical analyses.

## 5. Conclusions

This study was performed to evaluate alternative reagents and techniques for improving nucleic acid extraction, purification, and molecular testing for water samples. The developed lysis buffer, bead beating, inhibitor removal, and PCR facilitator procedure was shown to be effective in comparison to commercially available nucleic acid extraction kits. Using a challenging suite of concentrated source water and finished water from five drinking water utilities, the UNEX buffer-based extraction protocol was demonstrated to be generally effective for enabling sensitive detection of a diverse suite of viruses, bacteria and parasites by real-time PCR and RT-PCR. However, a limited number of replicate experiments were performed to study some aspects of the protocol, and some of the water matrices were associated with significant inhibition of the molecular assays, especially RT-PCR. Thus, while the reported nucleic acid extraction and molecular assay protocols should be useful for enabling sensitive detection of waterborne microbes, new technologies and additional research are needed to further improve such sample preparation methods for molecular testing of water samples.

## Appendix

Water quality data for the source and finished water samples are provided in Table A1. These data reflect the quality of the original water samples collected for the study, prior to concentration of the samples using ultrafiltration.

**Table A1:** Water quality data for water samples used to evaluate the water sample preparation procedure and alternative sample preparation techniques.

Utility	Water Type	pH	Turbidity (NTU)	TOC (mg/L)	DOC (mg/L)	Cond. (μS/cm)	Alkalinity (mg/L)
Utility A	Source	7.3	9.0	7.6	6.4	81.7	25.6
	Finished	8.1	0.40	3.5	4.3	20.5	1.7
Utility B	Source	7.8	0.30	16.7	13.5	1031	138
	Finished	7.6	0.08	12.9	11.3	1039	135
Utility C	Source	8.1	1.2	7.3	7.6	845	97
	Finished	8.1	0.14	5.2	4.7	856	96
Utility D	Source	6.6	0.96	3.1	2.4	63.0	8.0
	Pre-Finished	6.4	1.1	2.6	3.2	63.2	11.5
Utility E	Source	8.2	0.12	4.8	3.5	283	126
	Finished	7.9	0.50	4.6	4.0	326	137

^a^ ND = No data.
